# Tissue Fractions of Cadmium in Two Hyperaccumulating Jerusalem Artichoke Genotypes

**DOI:** 10.1155/2014/421249

**Published:** 2014-04-14

**Authors:** Xiaohua Long, Ni Ni, Zhaopu Liu, Zed Rengel, Xin Jiang, Hongbo Shao

**Affiliations:** ^1^Jiangsu Provincial Key Laboratory of Marine Biology, College of Resources and Environmental Sciences, Nanjing Agricultural University, Nanjing 210095, China; ^2^State Key Laboratory of Soil and Sustainable Agriculture, Institute of Soil Science, Chinese Academy of Sciences, 71 East Beijing Road, Nanjing 210008, China; ^3^School of Earth and Environment, The University of Western Australia, 35 Stirling Highway, Crawley, WA 6009, Australia; ^4^Key Laboratory of Coastal Biology & Bio-Resources Utilization, Yantai Institute of Coastal Zone Research (YIC), Chinese Academy of Sciences (CAS), Yantai 264003, China

## Abstract

In order to investigate the mechanisms in two Jerusalem artichoke (*Helianthus tuberosus* L.) genotypes that hyperaccumulate Cd, a sand-culture experiment was carried out to characterize fractionation of Cd in tissue of Cd-hyperaccumulating genotypes NY_2_ and NY_5_. The sequential extractants were: 80% v/v ethanol (F_E_), deionized water (F_W_), 1 M NaCl (F_NaCl_), 2% v/v acetic acid (F_Acet_), and 0.6 M HCl (F_HCl_). After 20 days of treatments, NY_5_ had greater plant biomass and greater Cd accumulation in tissues than NY_2_. In both genotypes the F_NaCl_ fraction was the highest in roots and stems, whereas the F_Acet_ and F_HCl_ fractions were the highest in leaves. With an increase in Cd concentration in the culture solution, the content of every Cd fraction also increased. The F_W_ and F_NaCl_ ratios in roots were lower in NY_5_ than in NY_2_, while the amount of other Cd forms was higher. It implied that, in high accumulator, namely, NY_5_, the complex of insoluble phosphate tends to be shaped more easily which was much better for Cd accumulation. Besides, translocation from plasma to vacuole after combination with protein may be one of the main mechanisms in Cd-accumulator Jerusalem artichoke genotypes.

## 1. Introduction

Cd is one of biotoxic metal elements, which has strong chemical activity and long-term toxicity and is relatively mobile in plants [1, 2]. It is also one of the major environmental pollutants. Moderate Cd contamination of arable soils can result in considerable Cd accumulation in edible parts of crops [3–5]. Cd can be present in plant tissues in concentrations that are nontoxic to crops but can contribute to substantial Cd dietary intake by humans [6].

Existing methods of cleaning up Cd-contaminated soils are expensive, such as mechanical removal and chemical engineering [7]. Comparatively, phytoextraction has a great potential in ameliorating Cd-contaminated soils because it is a cost-effective, environmentally friendly approach applicable to large areas [8, 9].

Plant resistance to metal toxicity stress includes avoidance and tolerance [10]. Avoidance frequently results in exclusion, whereas accumulation in plant tissues must be linked with internal tolerance mechanisms. These tolerance mechanisms might rely on metal being retained mainly in roots, with transport to photosynthetically active above-ground tissues impeded. In addition, tolerance may be underpinned by metals existing in nonactive (nontoxic) forms in plant tissues. Such nontoxic forms may, for example, include binding of metals in the cell wall or complexation with organic acid and proteins mostly in the vacuole [11].

We have previously reported that two Jerusalem artichoke genotypes, NY_2_ and NY_5_, when grown in Cd contaminated soils did not suffer from Cd toxicity, even though Cd concentration not only in roots but also in leaves and stems exceeded 100 mg kg^−1^ dry weight [12], which is the main feature of hyperaccumulators. Hence, NY_2_ and NY_5_ genotypes showed a potential to be used in phytoremediation of Cd-contaminated soils via phytoextraction. However, the research so far has mainly concentrated on Cd accumulation and plant physiological properties rather than on Cd chemical forms in Jerusalem artichoke genotypes. The Cd chemical forms in plant tissues are expected to be linked to Cd tolerance via detoxication mechanisms. An understanding of Cd-tolerance mechanisms in Jerusalem artichoke genotypes is a prerequisite for quick screening ofgermplasm for improved Cd accumulation and tolerance. This study was aimed at characterizing the distribution of Cd chemical forms in Jerusalem artichoke genotypes that hyperaccumulate Cd.

## 2. Materials and Methods

### 2.1. Plants

Two Jerusalem artichoke (*Helianthus tuberosus* L.) genotypes, NY_2_ and NY_5_, were selected from the Nanjing Agricultural University Experimental Station (“863 Program”) at Laizhou County in Shandong Province, China. Previous work [12] indicated that these two genotypes have the capacity to hyperaccumulate Cd.

### 2.2. Experimental Setup

The tests were carried out in a greenhouse at Nanjing Agricultural University (N32° 2′ 6.25′′, E18° 50′ 23.47′′), Nanjing, China. The average temperature throughout the test period was between 26.6 ± 4.4°C (daytime) and 22.0 ± 2.4°C (night), and the relative humidity was 61.5 ± 1.3% (daytime) and 68.0 ± 1.9% (night). Tuber slices with buds were germinated on sand moistened with 1/2 Hoagland nutrient solution in an incubator. The nutrient solution was replaced every second day. At trefoil stage, young plants were transplanted into porcelain pots. About one week later, Cd treatments were imposed (0, 2.5, 5.0, or 10 mg L^−1^ as CdCl_2_·2.5 H_2_O). Each Cd treatment was replicated in three pots, and two uniform plants were allowed to grow in each pot at a uniform spacing. Sampling was carried out after 3-week treatment duration.

### 2.3. Plant Sampling and Analysis

Roots were washed in deionized water, and then shoots and roots were separated, weighed, and used for sequential extraction to determine chemical forms of Cd [13, 14]. Briefly, 1 gram fresh leaf, stem, or root material was cut into pieces of 1-2 mm^2^, transferred into a beaker with 10 mL of extractant (Table 1), and kept at 25°C overnight (20–24 h) on a shaker [15]. The following day the solutes were saved, and the residues were extracted again overnight with the next extractant. In total, there were five sequential extractions.

The extracts were digested with a concentrated acid mixture of HNO_3_–HClO_4_ (3 : 1 v/v) and heated at 160°C for 5 h. After cooling, the extracts were diluted, filtered, and made up to 25 mL with 5% v/v HNO_3_. The Cd concentration in the extract was determined by inductively coupled plasma atomic emission spectroscopy (ICP-AES, IRIS Intrepid II XSP; Thermo Electron Company, USA). The analyses were carried out in triplicate.

### 2.4. Symbol Meanings

F_E_, F_W_, F_NaCl_, F_Acet_, and F_HCl_ show the amounts of the Cd-containing fractions extracted by ethanol, water, NaCl, acetic acid, and HCl, respectively.

### 2.5. Statistical Analysis

All statistical tests were performed using SPSS 13.0. Two-way ANOVA was used to determine the significance of genotype and Cd treatment effects on Cd forms. Mean treatment differences were separated by the least significant difference (LSD_0.05_) test if  *F*-tests were significant (*P* ≤ 0.05; Fisher's protected test).

## 3. Results

### 3.1. Effects of Cd Treatments on the Biomass and Its Components of Two* H. tuberosus* Genotypes

Even though the two Jerusalem artichoke genotypes showed good tolerance to Cd toxicity, NY_2_ showed some wilting in the high-Cd treatments. Compared to control, the Cd treatment (2.5 and 10 mg kg^−1^) decreased leaf, stem, root, and total biomass for both NY_2_ and NY_5_ (Table 2). In contrast, the 5 mg kg^−1^ Cd treatment increased the biomass and its components for NY_2_. In every Cd treatment, the biomass and its components of NY_5_ were lower than in the 0 mg kg^−1^ Cd supply, but there was no significant difference.

### 3.2. Chemical Forms of Cd in Plants

#### 3.2.1. Effects of Cd Treatments on Cd Chemical Forms of Roots in Two Jerusalem Artichoke Genotypes

As can be seen from Table 3, the distribution ratios raised with increased Cd supply in both NY_2_ and NY_5_. In control group, the difference between the five forms was not remarkable. In treatment group, the Fw ratio increased and was higher than the F_R_ ratio in NY_2_ and NY_5_. F_NACl_ occupied the most proportion in both two Jerusalem artichoke genotypes; secondly, F_Acet_ and F_E_ were the least.

There were some differences of the five main chemical forms in roots between NY_2_ and NY_5_. The Fw ratio was higher in NY_2_ than that in NY_5_. Water extracts the soluble Cd organic acid complex, Cd (H_2_PO_4_)_2_, which is poisonous and tends to cause harm to plants. The F_Acet_ ratio of high accumulator was higher than low accumulator, while the Fw ratio was lower. Ethylic acid extracts unsoluble CdHPO_4_, Cd_3_(PO)_2_, or Cd-phosphate complexes, which may be better for Cd accumulation in high accumulator.

#### 3.2.2. Effects of Cd Treatments on Cd Chemical Forms of Stems in Two Jerusalem Artichoke Genotypes

We can see from Table 4 that the distribution ratios were raised with increased Cd supply in both NY_2_ and NY_5_. Compared to the roots, every proportion of the five chemical forms in stems decreased greatly. Similarly, F_NaCl_ occupied the most proportion in both two Jerusalem artichoke genotypes; secondly F_Acet_ and F_R_ were the least. The Fw ratio was higher in NY_2_ than that in NY_5_. The F_Acet_ ratio of high accumulator was higher than low accumulator, while the Fw ratio was lower.

#### 3.2.3. Effects of Cd Treatments on Cd Chemical Forms of Leaves in Two Jerusalem Artichoke Genotypes

The distribution ratios were raised with increased Cd supply in both NY_2_ and NY_5_ (Table 5). Compared to stems, the five forms were further reduced in different degrees, especially F_NaCl_. F_Acet_ covered the most part, 38% and 41%, respectively, in NY_2_ and NY_5_.

The F_W_ ratio was the lowest in leaf instead of the F_R_ ratio in NY_2_. Ethanol extracts Cd-nitrate, Cd-chloride, and Cd-amino acid. The lowest F_NaCl_ ratio might be beneficial for protecting leaves because NaCl extracts mainly Cd-pectates, Cd-polypeptide, or Cd-protein.

## 4. Discussion

### 4.1. Cd Distribution in Cells of Roots

Cd concentration showed the same order (root > stem > leaf > tuber) and Cd accumulation in plant components showed the order of stem > leaves > roots for both NY_2_ and NY_5_ in previous work [12]. Cd chemical forms in plants were linked with Cd transporting activity, among which F_R_ and F_W_ were the strongest; secondly, F_NaCl_, F_Acet_, and F_HCl_ were the weakest [16]. Most of Cd enriched in roots after absorbing by plants and the amount transported up to shoots was usually a little [17]. Cd concentrated in roots which might be related to Cd that formed stable large molecule complex with protein, polysaccharide, ribose, and nuclein in roots, deposited [18], and then lightened poisoning to organs in shoots. Cd accumulation in roots is usually accredited to cell wall [19]. Cell wall is the first protective screen protecting cell protoplast from being harming by heavy metals. Cellulose, hemicelluloses, xylogen, and pectic substance which consist of cell wall have abundant active perssads, such as carboxyl, oxhydryl, and aldehyde group. A part of external Cd being passed through will combine with these perssads. It prevents large amount of Cd from going into plasma and reduces toxicity [16]. Especially in the condition of short time and low concentration, this tolerance system of the combination of Cd and cell wall is most important [20]. However, some scholars consider oppositely that the amount of Cd combined with root cell wall is much less than that in the cell [19, 21, 22]. Vacuole is a Cd accumulating place in higher plants, but not the main point, only in the condition of high Cd concentration [19, 23]. As can be seen from Table 3, the absolute advantage laid with the F_NaCl_ ratio, showing that Cd mainly adheres to protein. This is because that Cd has very strong affinity with protein or hydrosulphonyil in other organic compounds [24]. On the one hand, the combination of Cd and protein in plants can decrease the amount of free Cd, reducing its availability and mobility and avoiding harm to plants. Cd also may be combined with enzymes and functional proteins, disturbing their regular function and disordering physiological and biochemical metabolism [25]. Because of the higher F_w_ ratio and the lower F_Acet_ ratio, the amount of Cd is more poisonous and is transported more quickly in NY_2_ than that in NY_5_, with NY_2_ showing fewer biomass of roots in some degree and NY_5_ showing normal for the growth of 20 days.

### 4.2. Cd Transporting from Roots to Shoots

The transporting of Cd from roots to shoots and accumulated in shoots is a very complicated process. There are many studies on it [26]. It is usually considered that Cd absorbed by roots is transported to other components in plants through the xylem. Root metal ions go into root vascular bundle through endoderm and the inner casparian strip. Passing through casparian strip is difficult, so this translocation is mainly carried out in young roots in which casparian strip has not been formed completely [27]. Then mental ions may be transported up to shoots by transpiration. There are many reports on long-distance translocation and the system of Cd long-distance translocation in plants is controversial. The F_w_ and F_NaCl_ ratios were reduced substantially in both NY_2_ and NY_5_, so compared to control, stems did not suffer toxicity in NY_5_ while except when at the Cd concentration of 10 mg L^−1^ and the biomass of stem in NY_2_ decreased by small degrees.

### 4.3. Cd Accumulation and Distribution in Leaves

Cd in leaf cells mainly comes from the water translocation from vascular bundle to leaf tissue which indicates that transpiration plays an important role in heavy mental accumulation [19]. Similar to root cells, the combination of leaf cell wall and Cd decreases Cd concentration in cell sap, lightening toxicity to leaf cells. However, the interception of cell wall plays a secondary role and the main detoxication mechanism is in the vacuole [19]. There are rich small molecule substances, such as GSH, oxalic acid, histidine, citrate, and phosphoric acid in vacuole. Cd avoids contacting with organelle to realize Cd detoxication through chelation or laydown with those small molecule substances [28, 29]. Compared to other chemical forms, the F_Acet_ ratios have absolute advantage in both two Jerusalem artichoke genotypes showing that a considerable part of Cd tends to form insoluble phosphate in leaves; accordingly, the amount of free Cd which is poisonous becomes low (Table 5). Therefore, from the appearance, there is almost no remarkable difference between the biomass of treatment group and that of control group in NY_2_ and NY_5_ leaves. Previous studies have documented that Cd exists and transports in ion form in some certain plants [30, 31]. But in some Cd-accumulators, Cd existence is mostly in organic combination [31].

## 5. Conclusions

In summary, Cd toxicity and tolerance mechanism are most complex. Different plants, even different strains of the same plant or different ecological types, may show diverse Cd tolerance ability and mechanism. According to the previous study, compared with NY_2_, genotype NY_5_ may be a better candidate for phytoremediation of and biofuel production on Cd-contaminated soils. The present study implied that in high accumulator, namely, NY_5_, the complex of insoluble phosphate tends to be shaped more easily which is much better for Cd accumulation. Besides, translocation from plasma to vacuole after combination with protein may be one of the main mechanisms in Cd-accumulator Jerusalem artichoke genotypes.

## Figures and Tables

**Table 1:** Extractants and relevant extracted chemical forms of Cd.

Extract ion reagent	Code	Predominant forms of extracted Cd
80% v/v ethanol	F_E_	Cd-nitrate, Cd-chloride, Cd-amino acid complexes
Deionized water	F_W_	Soluble Cd-organic acid complexes, Cd(H_2_PO_4_)_2_
1 M NaCl	F_NaCl_	Cd-pectates, Cd-polypeptide, or Cd-protein complexes
2% v/v acetic acid	F_Acet_	Sparingly soluble CdHPO_4_, Cd_3_(PO)_2_, and/or other Cd-phosphate complexes
0. 6 M HCl	F_HCl_	Cd-oxalate

**Table 2:** Effects of different cadmium treatments on the fresh biomass of two *H. tuberosus *genotypes.

Genotype	Cd supply (mg L^−1^)	Leaves (g plant^−1^)	Stem (g plant^−1^)	Root (g plant^−1^)	Whole plant (g)
NY_2_	0	24.3^a^	10.7^ab^	23.3^a^	58.2^a^
	2.5	24.6^a^	11.1^ab^	21.1^a^	56.8^a^
	5	28.5^a^	13.0^a^	28.4^a^	69.9^a^
	10	15.4^b^	8.3^b^	11.1^b^	34.9^b^

NY_5_	0	29.8^a^	13.0^a^	25.8^a^	68.5^a^
	2.5	29.1^a^	12.8^a^	17.1^b^	58.9^a^
	5	30.1^a^	13.9^a^	20.9^ab^	64.9^a^
	10	27.5^a^	12.7^a^	16.9^b^	57.1^a^

Different letters within a column indicate the significant differences among the treatments (*P* ≤ 0.05,  *n* = 3).

**Table 3:** Fractionation of Cd in roots of two Jerusalem artichoke genotypes.

Genotype	Cd supply (mg L^−1^)	F_E_ (*µ*g g^−1^)	F_W_ (*µ*g g^−1^)	F_NaCl_ (*µ*g g^−1^)	F_Acet_ (*µ*g g^−1^)	F_HCl_ (*µ*g g^−1^)
NY_2_	0	11^a^	8.4^a^	11^a^	12^a^	8.3^a^
	2.5	41^c^	48^c^	223^a^	127^b^	35^c^
	5	38^b^	103^b^	655^a^	127^b^	54^b^
	10	62^b^	150^b^	1675^a^	329^b^	91^b^

NY_5_	0	3.7^c^	4.9b^c^	13^a^	7.9^b^	7.8^b^
	2.5	51^c^	36^c^	304^a^	113^b^	33^c^
	5	55^b^	83^b^	469^a^	259^ab^	63^b^
	10	80^b^	117^b^	1280^a^	387^b^	245^b^

Different letters within a row indicate significant differences among the fractions (*P* ≤ 0.05,  *n* = 3).

**Table 4:** Cd chemical forms of stems in two Jerusalem artichoke genotypes.

Genotype	Cd supply (mg L^−1^)	F_R_ (*µ*g g^−1^)	F_W_ (*µ*g g^−1^)	F_NaCl_ (*µ*g g^−1^)	F_Acet_ (*µ*g g^−1^)	F_HCl_ (*µ*g g^−1^)
NY_2_	0	7.0^a^	5.8^a^	6.6^a^	8.7^a^	6.4^a^
	2.5	17^c^	14^c^	126^a^	66^b^	35^b^
	5	18^b^	28^b^	278^a^	48^b^	34^b^
	10	22^b^	43^b^	315^a^	91^b^	57^b^

NY_5_	0	2.9^c^	5.3^b^	7.4^a^	6.5^ab^	6.4^ab^
	2.5	13^c^	13^c^	99^a^	54^b^	31^bc^
	5	16^a^	17^a^	195^a^	92^a^	47^a^
	10	32^c^	49^bc^	393^a^	135^b^	131^b^

Different letters within a row indicate the significant differences among the forms (*P* ≤ 0.05,  *n* = 3).

**Table 5:** Cd chemical forms of leaves in two Jerusalem artichoke genotypes.

Genotype	Cd supply (mg L^−1^)	F_R_ (*µ*g g^−1^)	F_W_ (*µ*g g^−1^)	F_NaCl_ (*µ*g g^−1^)	F_Acet_ (*µ*g g^−1^)	F_HCl_ (*µ*g g^−1^)
NY_2_	0	8.7^a^	5.2^a^	5.9^a^	9.4^a^	8.9^a^
	2.5	13^c^	6.9^c^	20^c^	70^a^	47^b^
	5	8.3^b^	12^b^	17^b^	44^a^	42^a^
	10	15b^c^	9^c^	20^b^	56^a^	45^a^

NY_5_	0	1.4^c^	5.3^b^	7.3^a^	6.5^ab^	7.5^a^
	2.5	7.4^b^	6.6^b^	14^b^	45^a^	47^a^
	5	10^b^	8.9^b^	14^b^	61^a^	78^a^
	10	15^b^	15^b^	41^b^	129^a^	112^a^

Different letters within a row indicate the significant differences among the forms (*P* ≤ 0.05,  *n* = 3).
